# 

**Published:** 1914-09-12

**Authors:** 


### CONTENTS.

PAGE

**The After-Care of the Wounded** ... ... ... 641
**Committees and the Shortage of Resident Officers** ... ... ... ... ... ... 642
**Hospital and Institutional News** ... ... ... 643
The King's Visit to the London Hospital—The King at St. Thomas's Hospital—Lord Kitchener's Surprise Visits — The Class of Case in Netley Hospital—New Secretary of the Queen's Hospital, Birmingham—Ejected Hospital Patients—The Families of Arrested Germans in London—Classifying Sanatoria Patients —The Unreliability of Cheap Thermometers—Temporary Wards at Newcastle Royal Infirmary—Hospital Work and Support in Newcastle—The King and his Wounded—Suggested New Name for the Metropolitan Asylums Board—The Reopening of Hertford County Hospital—Dublin Hospitals and the War—Developments at the London Hospital—A Hospital Chaplain and the Cholera Epidemic —Distinction for a Medical Superintendent—Infirmary Officer on Active Service—The Amalgamation of Manchester Unions—Naval Sick Landed at Aberdeen—Progress of the Workers' Fund at Cambridge—Hospital Wills—National Medical Union and the War—Panel Chemists and the Cost of Drugs—This Week's Drug Market.
**Physical Fitness and Military Efficiency** ... 649
Are the Medical Tests for Recruits too Severe?
**An Exposure of Quackery** ... ... ... ... 650
The Select Committee's Report on Patent Medicines.

PAGE

**Hospitals and the War** ... ... ... ... 651
Our Sick and Wounded Home Again.
Reception of the Wounded at St. Thomas's Hospital.
The Provincial Hospitals' Arrangements (From our Special Correspondents) Bristol, Cambridge—Chatham District: German Prisoners —Gravesend—Ipswich—Leicester: Arrival of Wounded—Leamington Spa—Manchester Men on Active Service—Middlesbrough—Norwich—The Temporary Wards at Newcastle—Sheffield: Wounded from Mons—Tunbridge Wells and District.
The Welsh War Hospital Scheme.
Arrangements in Edinburgh and Dundee: Dundee —Edinburgh: Arrival of Navy Men.
**The Institutional Library** ... ... ... ... 658
A Manual of First Aid.
A Book for the General Practitioner.
**Round the World** ... ... ... ... ... 659
Fellow-Workers and their Doings.
**Legal Notes for Institutional Workers** ... 660
Managers and Statutory Funds—A Judge on Medical Witnesses' Fees.
**Hospitals and their Contracts** ... ... 660
View of a London Secretary.
**Medical Appointments** ... ... ... ... 662
**Buildings Contemplated** ... ... ... ... 662
**Institutional Needs** ... ... ... ... ... 662
**The Institutional Worker** ... .. (Suppl.) 1
The Care of Mattresses and Bedding.
Water Softening.
Questions for August and September.
Questions Invited.
Inquiries and Answers: Employment and Training—The Sick and in Need—Acknowledgments.

